# Minutes of the Ohio State Dental Society

**Published:** 1874-02

**Authors:** C. R. Taft

**Affiliations:** Sec’y.


### Proceedings of Societies.

### MINUTES OF THE OHIO STATE DENTAL SOCIETY.

The eighth annual meeting of the Ohio State Dental Society was held in the Council Chamber at Columbus, Dec. 3d, 4th and 5th, 1873. The meeting was called to order by Dr. L. Buffett, President, and opened with prayer by the Rev. Mr. Hutchinson. The roll was called and the minutes of the previous meeting read, amended and, on motion of Dr. Horton, were received and filed.

The report of committees being called for, the Chairman of the Executive being absent, the Committee was granted further time.

Dr. Rehwinkle, Chairman of the Committee on Dental Ethics, reported that no cases had come to the knowledge of the Committee requiring its action.

The Membership Committee reported the name of Dr. W. H. Atkinson, of New York City, for honorary membership. On motion, he was unanimously elected. The name of Dr. D. G. French, of Pittsburg, Pa., was also presented for honorary membership. After some discussion as to the propriety of conferring this compliment too generally, Dr. French was elected an honorary member of this Society. Dr. Rehwinkle moved that Dr. N. W. Williams, a former member of this Society, Dr. Van Marter, Dr. G. W. Fields and Dr. C. M. Wright, all now practicing dentistry in Europe, be elected honorary members of this Society. The motion prevailed and they were declared duly elected. The names of Dr. Rollin Horton and Dr. R. R. Peebles, both of Cleveland, were presented by the Membership Committee for active Membership. On motion, the Secretary was instructed to cast an affirmative ballot for these names and they were declared elected.

A short recess was here taken.

Order being called, Dr. Horton offered the following:

*Resolved*, That his Honor, the Mayor of the city of Columbus, members of the Common Council, and other city officials, also clergymen and physicians, are hereby cordially invited to attend the meetings of this Society.

Resolution adopted.

The Membership Committee further reported the names of Prof. S. W. Dennis and Dr. Alex. Warner, of San Francisco, Cal., for honorary membership in this Society. A vote being taken, they were elected honorary members.

### 1ST DAY, AFTERNOON SESSION.

The Society re-assembled at 2 o'clock. Minutes of the forenoon session read and approved.

On motion of Dr. Rehwinkle, Dr. W. P. Horton was appointed a committee of one to notify the Mayor, members of the City Council, clergy and physicians of the city, of the invitation extended them by this body.

Dr. Watt stated that, as Dr. W. H. Atkinson, of New York City, was present, and might not be able to remain during the entire sittings of the Society, he would move that, in order to hear from Dr. Atkinson, the regular order be passed, and that the ninth subject for discussion—"Does the administration of the lime phosphates aid in the development of good teeth"—be taken up. Dr. Rehwinkle moved to amend, to make this topic the special order for discussion for to-morrow, at 10 o'clock, A. M. Amendment accepted and the motion prevailed.

The first subject for discussion was now taken up. "What are the benefits to be derived from a proper care of the deciduous teeth? Descriptions of methods of treatment." This subject was spoken to by Drs. Watt, Berry, Herriott, J. Taft, Von Bonhorst, W. H. Atkinson, W. P. Horton, Rehwinkel, Clippinger and Keely.

Dr. Clippinger moved that, when the Society adjourn, it be to 7 1-2, this evening. Carried.

The 1st subject was further discussed by Drs. Atkinson, Keely, Butler, Way, Gray and Herriott. The subject was very fully discussed in detail by the various members, occupying the entire afternoon until near the hour of adjournment.

Dr. Blount, Chairman of the Committee on Membership, reported the names of Drs. G. D. Billings, of Medina; F. H. Houghton, Columbus and F. O Jacobs, of Newark, for active membership. These persons, having been recommended, were balloted for and elected at the last meeting; but having failed to sign the Constitution, Dr. C. R. Taft moved that the motion of Dr. Rosson, passed at the last meeting, ordering the dropping of the names of all persons who failed to sign the Constitution and pay the initiation fee, be rescinded in so far as it applied to these candidates, as the omission was wholly inadvertent, and that they be permitted to sign the Constitution and become active members without a second ballot. The motion prevailed.

On motion, the Society adjourned to 7 1-2 o'clock P. M.

### 1ST DAY, EVENING SESSION.

The Society re-assembled at 7 1-2 o'clock, Dr. L. Buffett in the chair. Tne minutes of the afternoon session were read and approved.

Dr. Herriott moved that the second subject in the regular order be taken up; namely, "How much is the success of filling teeth due to the mere manipulation?

Dr. Waye read an essay on this subject, which was passed to the Publication Committee. The positions assumed by the author as well as the whole ground covered by the question were fully treated of, Drs. Atkinson, Watt, J. Taft, Way and Herriott speaking to the subject.

Dr. Watt stated that he had in his possession a letter from Dr. N. W. Williams, of Geneva, Switzerland, and asked if the Society desired to hear the letter. Dr. J. Taft moved that the letter be read at 9 o'clock, to-morrow morning.

The discussion of the second subject was continued by Drs. J. Taft, Rehwinkle and Watt. On motion, the Society adjourned to 9 o'clock, A. M., to-morrow.

2D DAY, MORNING SESSION.

Society called to order at 9 o'clock, A. M., and opened with prayer by the Rev. Mr. Laidlaw. Minutes of the previous session read and approved.

Dr. I. Williams made some remarks as to the manner of conducting the discussions in this Society.

Dr. Herriott moved that the 2d subject be passed and that the 3d subject in the order of discussion, that of "Observations on the methods of operating and materials used in filling teeth," be taken up. Dr. Herriott opened the discussion on this subject. Dr. Butler moved that in the discussion each speaker be allowed not to exceed fifteen minutes. Motion prevailed. The discussion was continued by Drs. J. F. Siddall, Clippinger, Berry and Gray.

The communication of Dr. N. W. Williams, of Geneva, Switzerland, being called for, Dr. Watt here read the letter of Dr. Williams addressed to the Ohio State Dental Society. It is as follows:

"GENEVA, SWITZERLAND,
Nov. 7th, 1873.

"THE PRESIDENT AND MEMBERS OF THE OHIO STATE DENTAL SOCIETY:—

"As the time of your next annual meeting draws near, I have a yearning of spirit to be with you as of yore; but, as that is an impossibility, I will, as a dutiful son to a fond parent, write and give an account of myself.

"The one great loss I have sustained, in leaving my native land, is the meetings of the many good associations which, with the dental journals, have been the means of placing our profession so prominently before the world, and of causing the name of "*American dentist*" to be respected and honored in Europe as the name of *dentists* is not. Europeans look upon their own dentists in the same light they do their barbers, and respect them about as much. But the high standard which we have attained at home compels them to honor our profession as they do the others. But to what base uses some things are brought! The high reputation which American dentists have, and the superior skill which they possess, are made a platform for continental quacks to rest their pretensions upon. In every city, Germans, French, Italians, who speak scarcely a word of English, advertise themselves as "American dentists." One we have, who sports D. D. S. on his sign, who studied medicine here and could not pass an examination, but went to Philadelphia, and returned with his diploma, *and he fills front teeth with black amalgam.* What a disgrace to our fair name, that our colleges should send out such miserable specimens ! Is it for the money, or is it to show a long list of graduates ? If these institutions will send out such at home, for heaven's sake let them spare us abroad, where a diploma from a college means something. The standard of even Amerlcan dentistry is low in this country. Very few are operating up to the standard of the profession at home. So many are tempted by the almighty dollar to a quantity of business, regardless of the quality. We need the stimulus of associations ; for where would you be in America, if it was not for your associated efforts ? and to that end a few of us in this part of Europe met on the "glorious Fourth," and organized an American dental society ; and, as you will see from the enclosed circular, we will have a meeting for discussion on the 27th, to which we have invited all reputable American dentists whose addresses we could learn, and have invited them to meet with us, and we hope to be able to give a good report of ourselves.

"Wishing you all God's speed, I am, very truly,

"Your co-worker,

"N. W. Williams, D. D. S."

The hour having arrived for the reading of papers and discussions of the 9th subject, "Does the administration of the lime phosphates aid in the development of good teeth ?" the subject was opened with the reading of an essay by Dr. Porter. Discussion was opened by Dr. Watt and continued by Drs. Atkinson, Herriott, Porter, I. Williams, Butler, Way, Cressinger and J. Taft.

The Committee on Membership reported the following names for membershfp : R. L. Evans, Toledo ; L. G. Dills,

Greenville ; J. Rabe, Upper Sandusky ; H. J. Cressinger, Ashland ; J. C. Scott, Lancaster ; R. D. Waite, Coshocton ; J. F. Dennis, Washington C. H.; R. P. Moon, Van Wert ; C. E. Ruhl, Findlay.

On motion, the Secretary was instructed to cast the ballot for these candidates, and they were declared duly elected.

Dr. Herriott, Chairman of the Judiciary Committee, stated that his Committee was prepared to report. The report, being in order, was read, which showed four hundred and thirty-three dentists practicing in the State, classified as follows : ninety-nine are graduates, one hundred and forty-two have certificates from the State Board of Dental Examiners and one hundred and ninety-two have neither diplomas nor certificates.

The report was accepted and referred to the Publication Committee.

On motion of Dr. Berry, the report, together with the list of names and professional standing of each, was ordered printed and a copy sent to all the dentists in the State.

On motion of Dr. J. Taft, an appropriation of one hundred dollars was made to defray the expenses of the Judiciary Committee.

Dr. J. Taft moved that, when this Society adjourn, it be to 7 1-2 o'clock, this P. M. Carried.

Dr. H. A. Smith moved that the hour of 9 o'clock this evening be set apart for the election of officers to serve for the ensuing year. Motion carried.

The death of Dr. B. F. Rosson, a member of this society, being referred to by Dr. Keely, on motion, a committee of three, consisting of Drs. Keely, Williams and Watt, was appointed to give expression to the sense of this Society in reference to the death of our esteemed professional brother.

On motion, the Society adjourned to 7 1-2 o'clock, P. M.

### 2D DAY, EVENING SESSION.

The Society re-assembled at 7 1-2 o'clock. The minutes of the previous session were read and approved.

The fourth subject in the order of discussions was taken up —"Why has not dentistry received the general recognition, as a specialty of medicine, to which it is entitled?" Dr. J. F. Siddall read an essay on this subject and the topic was discussed by Drs. J. H. Warner, Atkinson, J. Taft, H. A. Smith and Moon.

The Committee on Membership reported the names of the following persons for membership: T. G. Bristor, Mansfield and T. F. Hnnter, Cambridge. On motion, the Secretary was instructed to cast the ballot for these candidates and they were declared duly elected.

The hour for the election of officers having arrived, on motion of Dr. I. Williams the House resolved itself into Committee of the Whole for the purpose of nominating officers for the ensuing year. Dr W. P Horton was called to the chair.

For President, Drs. H. A. Smith, I. Williams and C. R. Butler were nominated; for 1st Vice-President, Drs A. A. Blount, Harroun and Clippinger were nominated; for 2d Vice-President, Drs. Porter, Beauman and H. M· Reed were nominated; for Recording Secretary, Drs. Porter, Butler and C. R. Taft; for Corresponding Secretary, Drs. Butler, Smith and Jennings were nominated; for Treasurer, Drs. Williams, Horton, Herriott and Keely were nominated.

Motion to close the nomination was made and carried.

Dr. J. Taft moved that the Committee now arise and that the Society proceed to the election of officers. Motion prevailed.

The Chairman, Dr. Horton, now made the report of the Committee.

The President resumed the chair and appointed Drs. Porter and J. H. Warner as tellers. The Society then proceeded to ballot.

For President, Dr. H. A. Smith was elected; for 1st Vice-President, Dr. A. A. Blount was elected: for 2d Vice-President, Dr. J. B. Beauman was elected; for Rec. Sec'y, Dr. J. M. Porter was elected: for Corres. Sec'y, Dr. D. R. Jennings was elected; for Treas., Dr. G. W. Keely was elected.

On motion, these elections were all made unanimous.

Two vacancies having occurred in the State Board of Dental Examiners, by expiration of term of service of Drs.

W. P. Horton and M. Decamp, on motion, the Society proceeded to fill these vacancies. For these offices the following nominations were made: Dr. W. P. Horton, M. DeCamp and Dr. C. R. Butler. A ballot being had, Dr. W. P. Horton and Dr. C. R. Butler were declared elected members of the State Board, to serve the term of three years.

On motion, the Society adjourned to 9 o'clock, A. M. tomorrow.

THIRD DAY, MORNING SESSION,

The Society re-asembled at 9 o'clock, Dr. Buffett in the chair. Minutes of last session read and approved.

Dr. Rehwinkle moved that the discussion of the fourth subject be closed, and that the fifth subject be passed, and that the sixth in the order be taken up, namely: "Conservative treatment of exposed or diseased pulps. What are the best materials for filling the roots of teeth?" The motion prevailed.

Dr. Taft said as there were some committees to report he would move that they be heard at this time. Carried. Dr. I. Williams, the retiring Treasurer, made his report, as follows;

"The Treasurer begs leave to submit the following report:

| | |
|---|---|
| Received from former Treasurer . . . . | $1496.92 |
| " on Dues . . . . . . . . . . | 114.00 |
| " " Initiations for Dec. 3 and 4, 1873 | 45.00 |
| Total receipts for the year . . . . . | 1655.92 |

1873.

| | | |
|---|---|---|
| Apr. 5, | Paid to W. P. Horton, on order . | $24.50 |
| " 24, | " " F. H. Rehwinkel, Chairman Com. on Rubber. . . . | 500.00 |
| " " | " " H. A. Smith, Chairman of Com. on Enforcement of Dental Law . . . . . | 300.00 |
| Dec 4, | " " W. M. Herriott, Chairman of Judiciary Committee . . | 100.00 |
| | | 924.50 |

Leaving a bal. in the hands of the Treasurer of $731.42 plus the accruing interests on bank deposits.

All of which is most respectfully submitted.

I. WILLIAMS,
*Treas.*"

The sixth subject for discussion was now taken under consideration. Dr. J. F. Siddall stated his views and practice and was followed by Dr. Williams, who fills the roots of teeth with tin foil, believing it to be one of the best materials in use for this purpose. He afterward fills the pulp cavity and tooth with gold. Dr. Butler gave his views as to filling roots of teeth, greatly preferring gold in all cases, and detailed his method of operating at some length.

Prof. Atkinson followed, who gave his method of treating and filling roots. Dr. Herriott preferred to hear the treatment necessary to preserve the pulps rather than the method of filling roots. Dr. Butler stated that in his former remarks he did not mean to be understood as ignoring the conservative treatment of exposed pulps.

Dr. J. Taft said that the importance of preserving all pulps could not be overestimated, as the preservation of the teeth for any great length of time depended upon the vitality of the pulp. Dr. Howe followed recommending chloride of zinc treatment.

Dr. Rehwinkle called attention to the difference between oxide of zinc, which is neutral, and hydrochloride, which is an escharotic, and gave some of the different modes of application of these agents to pulps of teeth, as well as other methods of treatment. On the conclusion of Dr. Rehwinkle's remarks, Dr. Herriott moved that the discussion on this subject close and that the Society have the report of the Committee on Dental Patents. Carried: whereupon Dr. Rehwinkle, Chairman of the Committee, made a lengthy verbal report, which, on motion, was accepted.

On motion of Dr. Herriott, the action of the Dental Paten Committee and their disposition of the funds placed at their disposal were approved by the Society.

On motion, Drs. J. M. Porter and J. F. Siddall were appointed an Auditing Committee to whom all bills be referred.

Dr. J. Taft moved that the officers elect be now installed. Carried.

The President appointed Dr. Keely to conduct the President elect to the chair. Dr. Smith, on taking the chair, thanked the Society for the honor they had conferred upon him and complimented the Society, remarking that he knew of no similar organization which had been so efficient or industrious as this Society, and for these reasons he regarded it a mark of distinction to be called to preside over it.

Dr. Buffet now read his retiring address.

President Smith announced the following standing committees to act for the coming year:

| | |
|---|---|
| Executive Committee | F. H. Rehwinkle,<br>J. H. Warner,<br>C. H. Harroun. |
| Membership " | I. Williams,<br>E. J. Waye,<br>L. Buffett. |
| Publication " | J. Taft,<br>M. DeCamp,<br>D. W. Clancey. |
| Judiciary " | A. A. Blount,<br>C. R. Taft,<br>D. R. Jennings. |

On motion, the Society adjourned to 1 1-2 o'clock, P. M.

THIRD DAY, AFTERNOON SESSION.

Society re-assembled at 1 1-2 o'clock, the President, Dr. Smith, in the chair. Minutes read and approved.

On motion of Dr. J. Taft, the following persons were appointed delegates to the American Dental Association, to be held in Detroit, Aug. 4th, 1874, viz: R. L. Evans, Sam'l Clippinger, J. F. Siddall, Geo. Watt, C. R. Sabin, C. R. Taft, A. F. Emminger, P. A. Palmer, J. F. Dennis, J. H. Warner, L. M. Gray, D. W. Clancey, H. M. Reed and J. C. Scott.

On motion, the President and Secretary were empowered to add to the number of delegates and appoint alternates.

Dr. J. H. Warner offered the following resolution, which, on motion, was adopted:

*Resolved*, That the Secretary of the Examining Board be requested to present to this body a list of all members of this Society who have passed the Board without paying for their certificates and thereby been admitted to membership in this Society.

Dr. Horton, Secretary of the State Board of Dental Examiners, submitted his report as follows:

"To the Ohio State Dental Society:

Gentlemen:—Your Board of Examiners begs leave to report that since December 6th, 1872, at the date of the last report three meetings of the Board have been held.

The first, at the city of Cincinnati, on the 4th and 5th days of March last. At that meeting but one candidate presented himself for examination. The applicant was not found qualified.

The second meeting was held on the twelfth day of June, at Put-in Bay, at which time but one candidate presented himself, to whom, after due examination, a certificate was granted.

Some special examinations in writing were made by members of the Board and reported for the full Board's decision.

The third meeting was held in the city of Columbus, commencing on the third day of December, 1873, at twelve o'clock. At this meeting there appeared twelve applicants, to whom, after being fully examined, certificates were granted to eight.

It will be observed that the number of applicants has greatly fallen off since last year. This comes from the fact that the Ohio Legislature at its last session was for some reasons induced to modify the law under which this Board was created. As the law now stands, it requires a practice of only five years in this State to be exempt from its provisions.

Your Board does not feel like making any recommend tions as to the future, but will leave the question for you wisdom to determine what—if anything—shall be done in the premises to restore the law to its former usefulness.

Your Board has learned of your action in filling the vacancies caused by the expiration of the term of Drs. DeCamp and Horton. While the members can not question the wisdom of your selection, yet they very much regret that they are obliged to part company with Dr. DeCamp. Having been one of their number from the organization, and being an efficient member and a kind-hearted Christian gentleman, his loss can but be felt. While we regret his absence, we most cordially welcome his successor, Dr. C. R. Butler.

You will herewith find a report of finances, from Dec. 6th, 1872, to Dec. 6th, 1873:

The Ohio State Dental Society

In acct. with Examining Board,

| | | |
|---|---|---|
| 1873 | | Dr. |
| March 5 | To Expenses of Meeting, | $150.00 |
| June 12 | " " " " | 90.00 |
| Dec. 6 | " " " " | 188.00 |
| | | 428.10 |
| | By Cash from Certificates | 200.00 |
| | | 228.00 |

W. P. Horton,

Dec. 6th, 1873. *Treas.*

We would further report that the following have received certificates since the last annual meeting: L. C. Kelsey, Elyria, Loraine Co.; H. H. Whessen, Beverly, Washington Co.; N. B. Achison, Youngstown, Mahoning Co.; A. J. Grosvenor, Troy, Miami Co.: Frank J. Dawson, Lancaster, Fairfield Co.; Robert D. Wait, Coshocton, Coshocton Co.; Herod F. Hunter, Cambridge, Gurnsy Co.; Henry J. Cressinger, Ashland, Ashland Co.; Frank H. Houghton, Columbus, Franklin Co.

Respectfully submitted,

W. P. Horton, *Sec'y*, J. Taft, *Pres.*"

On motion of Dr. Herriott, the report was accepted and adopted.

The Auditing Committee, through Dr. Porter, reported bills to the amount of ($133.24) one hundred and thirty-three dollars and twenty-four cents, which were ordered paid.

Dr. Rehwinkle moved that a vote of thanks be tendered the Mayor and City Council of Columbus for their generosity in allowing the Society the use of their Hall without charge, and that the same be published in the city papers Carried.

On motion, a vote of thanks was tendered Dr. Emminger for the efficient manner in which he has performed his duty as a member of the Executive Committee.

A vote of thanks was also tendered the clergy of the city for their services.

Dr. Keely, Chairman of the Committee appointed to give expression to the sense of this Society on the death of Dr. Rosson, presented the following:

"Died, at Troy, Ohio, March 4th, 1873, B. F. Rosson, D. D. S., aged 48 years.

In these repeated visitations of sadness, by which we are called to part with a brother we cherish, we recognize in this our affliction the hand of a Father who doeth all things well, and before whom we should bow in humble submission.

In his death we mourn the loss of a faithful friend and brother, one who always had the interest of the profession at heart and who endeared himself to all by his genial disposition, conspicuous talents and true moral worth. We tender our deepest sympathies to his family, though conscious how little they will avail.

*Resolved*, That a copy be sent the family of the deceased, as also a copy to the REGISTER for publication.

GEO. W. KEELY,<br>

GEO. WATT,<br>

I. WILLAMS.<br>

} Committee"

On motion of J. H. Warner, the report was accepted and adopted as the sense of this Society.

On motion of Dr. Rehwinkle, the Society adjourned to the first Wednesday in Dec., 1874. C. R. Taft, *Sec'y.*

